# Catquest-9SF questionnaire: validation of the Portuguese version using the Rasch analysis

**DOI:** 10.5935/0004-2749.20230014

**Published:** 2022-01-31

**Authors:** Helmer Magalhães Antunes, Lívia de Castro Magalhães, Galton Carvalho Vasconcelos, Bruno Lovaglio Cançado Trindade, Ana Claudia Moreira Gonzaga, Renata Pereira Gonçalves Antunes

**Affiliations:** 1 Universidade Federal de Minas Gerais, Belo Horizonte, MG, Brazil.; 2 Visão Instituto, Conselheiro Lafaiete, MG, Brazil.; 3 Instituto de Olhos Ciências Médicas, Belo Horizonte, MG, Brazil.; 4 Instituto de Oftalmologia Cançado Trindade, Belo Horizonte, MG, Brazil.; 5 Faculdade Santa Rita, Conselheiro Lafaiete, MG, Brazil.

**Keywords:** Cataract extraction, Sickness impact profile, Visual acuity, Surveys and questionnaires, Quality of life, Extração de catarata, Perfil do impacto da doença, Acuidade visual, Inquéritos e questionários, Qualidade de vida

## Abstract

**Purpose:**

The aim of this study was to validate the Portuguese version of Catquest-9SF through its application in a native Brazilian population with cataracts and to determine the correlation of the questionnaire scores with preoperative visual acuity.

**Methods:**

A prospective study was conducted to validate the Catquest-9SF questionnaire, which was translated and back-translated, generating a final version in Portuguese. A total of 120 Brazilian patients awaiting cataract surgery were recruited to answer the questionnaire and to document their preoperative data and visual acuity. The Rasch analysis was used to assess the instrument’s psychometric properties.

**Results:**

The Portuguese version of Catquest-9SF demonstrated an acceptable adjustment of the items (item fit statistics ranging from 0.7 to 1.3), unidimensionality (principal component analysis), and good organization in the item response categories (thresholds of the categories: -2.79, 0.57, and 2.22, respectively). The questionnaire contains items with stable relationships if considered at the same level of visual impairment in the comparison between the two groups (absence of differential item functioning). The separation of people (person separation index, 3.07) was adequate. The visual acuity in the logarithm of the minimum angle of resolution (logMAR) in the best eye with the best optical correction showed a statistically significant correlation with the Catquest-9SF logit score (r=0.282 and p=0.004).

**Conclusions:**

The Portuguese version of Catquest-9SF presents evidence of validity and reliability, in addition to being linguistically and culturally understandable for Portuguese-speaking patients born in Brazil. The questionnaire is easy to understand and quick to apply, as it could adequately estimate the subjective visual functioning in patients with cataracts. We found a significant correlation between visual acuity and the questionnaire score.

## Abstract (pt)

**Objetivos:**

Validar a versão em português do Catquest-9SF através de sua aplicação em uma população nativa do Brasil com catarata e determinar a correlação da pontuação obtida no questionário com a acuidade visual pré-operatória.

**Métodos:**

Realizou-se um estudo prospectivo para validação de questionário. O Catquest-9SF foi traduzido e retro traduzido gerando uma versão final em português. Um total de 120 pacientes brasileiros que aguardavam realização de cirurgia de catarata foram recrutados para responder ao questionário e para documentação de dados pré-operatórios e acuidade visual. Análise Rasch foi utilizada para acessar as propriedades psicométricas do instrumento.

**Resultados:**

A versão em português do Catquest-9SF demonstrou ajuste aceitável dos itens (item fit statistics variando entre 0,7 e 1,3), unidimensionalidade (Principal Component Analisis) e boa organização nas categorias de resposta dos itens (limiares das categorias: -2,79; 0,57; 2,22). O questionário contém itens com relação estável, se considerado um mesmo nível de deficiência visual, na comparação entre dois grupos (ausência de *differential item functioning*). Existe adequada separação de pessoas (Person Separation Index 3,07). A acuidade visual em LogMAR no melhor olho com melhor correção óptica mostrou correlação estatisticamente significativa com a pontuação em logit do Catquest-9SF (r=0,282 e p=0,004).

**Conclusões:**

A versão em português do Catquest-9SF apresenta evidência de validade e confiabilidade, além de ser linguística e culturalmente compreensível para pacientes de língua portuguesa naturais do Brasil. Trata-se de questionário de fácil entendimento e rápida aplicação, sendo capaz de estimar de maneira adequada o funcionamento visual subjetivo em pacientes com catarata. Existe correlação significativa com a acuidade visual e a pontuação obtida no questionário.

## INTRODUCTION

According to the most recent assessment, cataracts are responsible for 51% of all cases of global blindness, which represents approximately 20 million people worldwide^(1)^. In recent years, cataract surgery has been undergone a huge technological development, including the techniques used in the procedure. As a result, it has reduced surgical time, lower complication rates, and decreased operating costs and provided high recovery predictability. Today, the phacoemulsification technique using a foldable lens implant is the most common ophthalmic surgery for cataract correction in the world^(2,3)^, being currently used as a refractive procedure and more frequently indicated than in the past^(4,5)^.

Catquest-9SF is a specific instrument created to assess personal visual quality of life perception in patients with cataracts. It is aimed at measuring visual problems perceived by patients in their daily lives and consists of nine items, of which seven assess the performance in activities of daily living and two assess the patient’s general perception of difficulties, in addition to their visual satisfaction. Initially created with 17 items in Sweden, Catquest was used to measure self-reported visual function changes 6 months after cataract surgery and to compare them with data collected before surgery. Since then, this instrument has been adapted to increase its validity and reliability. In 2009, the authors applied the item response theory, specifically the Rasch model, to this questionnaire, resulting in Catquest-9SF, which is comprised of nine items^(6)^.

From a clinical point of view, Catquest-9SF has the advantage of being an instrument of rapid application, with high graduation precision and sensitivity to changes caused by cataract surgery. Furthermore, the instrument is validated in terms of its psychometric properties by using the item response theory (specifically the Rasch model), which is part of a modern group of psychometric models to construct, validate, and evaluate measurement instruments and health outcomes. Catquest-9SF has already been validated for other languages such as English, Chinese, Spanish, German, Australian English, Italian, Dutch, Swedish, Slovak, and Malay. Owing to its peculiarities, Catquest-9SF is currently used in research in the European and Australian continents, being an important instrument for measuring visual quality after cataract surgery in multicenter analyses^(7,8,9,10,11,12)^.

Up to the present study, only two known tests/questionnaires have been validated and culturally adapted into the Portuguese language to measure visual quality of life, namely the National Eye Institute-Refractive Error Quality of Life (NEI-REQoL) instrument^(13)^, specifically used for refractive surgery candidates, and the short version of the Visual Function Questionnaire (VFQ-25), developed by the US National Eye Institute for the assessment of quality of life in several visual conditions^(14)^. However, still no specific questionnaire has been validated for cataract surgery that has psychometric qualities adapted to assess the quality of life in these cases. The objective of this study was to translate and cross-culturally adapt the Brazilian version of Catquest-9SF through its application in a Brazilian population with cataracts. It was also developed to assess the correlation between visual acuity in the best eye and the questionnaire score.

## METHODS

### Transcultural translation and adaptation

The following are the steps in the development of the Brazilian version of Catquest-9SF^(13)^:

The first step is called “forward translation,” in which two independent translators, one of whom was a Brazilian ophthalmologist fluent in English, performed the translation into Portuguese, creating two separate versions.
Subsequently, with the help of an arbitrator, the two initial versions were compared, producing a single common translation in Portuguese (Version X).
The third stage is called “back translation,” in which two new translators, both native speakers of English who were fluent in Portuguese and had no contact with the original version, translated version X into English, generating versions Y and Z.
An evaluation committee formed by two ophthalmologists fluent in both languages and the research author compared all versions (X, Y, and Z), generating a reconciled translation.
This version was used in a pilot test with five patients diagnosed as having cataract who were not part of the sample in this study to assess the comprehension of the questions and any inconsistencies. After the last adjustments, the final Portuguese version of Catquest-9SF was obtained (Table 1).

**Table 1:** Portuguese version of Catquest-9SF

Item
1. Você acredita que sua visão no momento esteja de alguma forma causando dificuldades nas suas atividades diárias?
2. Você está satisfeito ou insatisfeito com sua visão no momento?
Você apresenta dificuldades em realizar as tarefas abaixo por causa da sua visão? Se sim, o quanto? (sim, dificuldade muito grande; sim, grande dificuldade; sim, alguma dificuldade; não, nenhuma dificuldade)
3. Lendo um jornal impresso
4. Reconhecendo o rosto das pessoas que você encontra
5. Vendo o preço das mercadorias no supermercado
6. Enxergando obstáculos em pisos irregulares (ex. Paralelepípedos
7. Enxergando para fazer artesanato, costura ou outros trabalhos manuais
8. Lendo legendas em filmes na televisão
9. Enxergando para praticar alguma atividade de lazer que você tenha interesse

### Study participants

After the Portuguese translation process was completed, 120 patients on the waiting list for cataract surgery in the city of Conselheiro Lafaiete, MG, Brazil, were invited to participate in the validation study. After voluntary acceptance to participate, all the patients completed the questionnaire through interviews conducted by a trained nurse. The subsequent preoperative assessment included evaluation of visual acuity with better correction, biomicroscopy, eye pressure, fundoscopy, and biometric calculation.

The exclusion criteria included patients with difficulty in understanding and communicating in spoken or written Portuguese for any reason, with severe ocular comorbidities, and who needed combined surgical procedures in addition to phacoemulsification. The study was approved by the research ethics committee of the Federal University of Minas Gerais.

### Rasch analysis

The Rasch analysis was used to evaluate the Brazilian version of Catquest-9SF with the Winsteps 2020 software (Version 4.5.2) by using the Andrich rating scale model^(14)^.

By definition, the Rasch model is based on the understanding that the interaction between an item and a subject depends only on the subject’s ability (person’s measurement; for example, the extent to which the person has the ability being tested) and the item difficulty (item calibration; for example, the level of difficulty of the item). Ability and difficulty are mathematically represented through the Rasch analysis using the same interval scale, or logit (log odd unit), and can be compared. By showing the relationship between the subjects’ ability and the item difficulty, the model provides a detailed information on the measurement properties of the scale^(15,16)^. This, however, is only possible if the items collaborate to measure a one-dimensional construct, which must be confirmed to support the validity of the scale (reference).

By using logarithmic transformations, the Rasch model is essentially a probabilistic theoretical model to which the collected data are compared^(17)^. Conventionally, “logit” is attributed to the average difficulty of an item. Considering the “people” category, the logit measurement indicates how a person is more skilled than another (for example, whether a person has greater visual ability than another). Considering the “item” category, the logit calibration indicates how difficult an item is than another (for example, is reading a printed newspaper more difficult than recognizing faces of people you meet?).

Each Catquest-9SF item is scored using a scale of four categories numbered in such a way that patients with high levels of visual impairment, theoretically, would choose categories with higher scores (3 or 4, greater difficulty/dissatisfaction) and lower levels of disability and, therefore, would choose categories with lower scores (1 or 2)^(7)^. Similarly, items that address the performance of more-complex activities would receive higher scores by people with greater visual impairment. If all items work in this manner, they are combined to measure the same construct. However, in real situations, patients with poor visual ability unexpectedly have low scores in complex items and vice versa. This usually occurs when the item is poorly formulated, raising questions, or when it does not measure the same construct and does not fit with other items. The test validity is threatened when many items do not fit the model^(15,17)^.

The Rasch model provides the adjustment indicators infit and outfit mean square (MNSQ), which signal the unexpected behavior of the item. An MNSQ value between 0.7 and 1.3 is considered an indicator of unidimensionality. Items outside this range can be revised or removed to improve fit^(14,16)^.

Another important indicator generated by the software is the verification of the principal component analysis (PCA) residuals. This indicator is used in association with adjustment statistics (infit) to verify the unidimensionality of the measured construct. The PCA groups related the items in the main component, and the variance explained by the measurements should be at least comparable with that of the model (>50%). An unexplained variation in the first contrast of residuals (>2.0 eigenvalue units) suggests the existence of a secondary trait captured by the instrument^(14)^.

An important aspect of the analysis is to verify the adequacy of the item score scale. The threshold is the transition point between two response categories on a Likert polytomous scale. Therefore, a logical ordering of response categories to the same item is expected; that is, as the degree of visual impairment increases, people tend to score in the higher categories. A threshold disorder occurs in the absence of this logical ordering, signaling that the categories are not properly used. Associated with the thresholds, the probability of response curves, which represent the interaction between the ability level and response probability in each category, must be observed^(7,16)^.

The overall accuracy can also be measured using the person separation index (PSI), which represents the ability of a set of items to “separate” or differentiate the ability of different groups of subjects. A PSI of 3.0 indicates that the items separate people into at least three skill extracts, which represents a good level of separation^(7,8,14,15)^.

Finally, the Rasch model starts from the assumption that the behavior of an item is only a result of the level of ability (visual impairment) of the subjects who respond to it. Characteristics such as sex and age should not influence the item behavior. On the other hand, item calibration variations according to the specific characteristics of the subjects signal item differential response, also called “differential item functioning (DIF),” which impacts validity. A DIF value >1.0 logit is considered significant, indicating that the item has no stable relationship at the same level as the latent trait, when two groups are compared^(8,14,15,16)^.

### Correlation between Catquest-9SF score and visual acuity

To examine the ability of the questionnaire to discriminate groups with different levels of visual acuity, the correlation between best-corrected visual acuity in logMAR and the Catquest-9SF score was analyzed.

## RESULTS

### Sample characteristics

Of the 120 evaluated volunteers, 101 patients participated in the validation process. Difficulty in understanding and communicating in spoken or written Portuguese accounted for 79% of the 19 patients excluded. The mean (±standard deviation) age was 70.26 ± 9.003 years, and 53.5% of the patients were female (Table 2).

**Table 2:** Characteristics of the study population

Characteristic	n (%)
Sex	
Male	47 (46.5)
Female	54 (53.5)
Age	
≤70 years	48 (47.5)
>70 years	53 (52.4)
Previous cataract surgery	
Yes	11 (10.9)
No	90 (9.1)
Occupation	
From home/retired/pensioner	94 (94)
Worker or unemployed	7 (7)
Comorbidities	
Diabetes	21 (20.8)
Hypertension	79 (78.2)
Visual acuity in the best eye (LogMAR)	
Reach	0.1 even hand movement
Median (quartiles)	0.6 (0.4/0.6)

LogMAR= logarithm of the minimum angle of resolution

### Questionnaire validity: unidimensionality

In general, the infit and outfit MNSQ values were between 0.7 and 1.3 (Table 3), indicating an acceptable adjustment of the items considering the expectation of the model. The PCA explained 69.3% of the observation variances, suggesting no evidence of multidimensionality in the scale. The unexplained variance in the first contrast was 1.77 eigenvalue units, showing no evidence of a second dimension captured by the scale.

**Table 3:** The Catquest-9SF questionnaire with item difficulty calibration, infit and outfit mean square, and standardized fit statistics

Item	Item calibration	SE	Infit		Outfit	
			MNSQ	ZSTD	MNSQ	ZSTD
1. Do you experience your present vision causing you difficulties in any way in your daily life?	-0.31	0.18	0.92	-0.47	1.14	0.83
2. Are you satisfied or dissatisfied with your present vision? For the 7 difficulty items: Do you have difficulty with the following activities because of your vision? (yes, extreme difficulty; yes, great difficulty; yes, some difficulty; no, no difficulty)	-1.61	0.18	1.24	1.57	1.83	2.75
3. Reading text in the newspaper	-0.63	0.18	0.82	-1.2	0.87	0.65
4. Recognizing faces of people you meet	1.70	0.19	1.15	1.09	1.13	0.7
5. Seeing product prices in the supermarket	-0.10	0.18	0.63	-2.81	0.56	-2.95
6. Seeing to walk on uneven ground	1.22	0.18	1.07	0.55	0.98	-0.04
7. Seeing to do needlework and handicraft	-0.72	0.18	1.1	0.67	1.05	0.3
8. Reading text on television	-0.33	0.18	0.87	-0.84	0.9	-0.49
9. Seeing to carry out a preferred hobby	0.78	0.19	1.17	1.12	1.13	0.74

MNSQ= mean square; ZSTD= standardized fit statistic; SE= standard error.

### Item score scale performance

**Figure 1:**
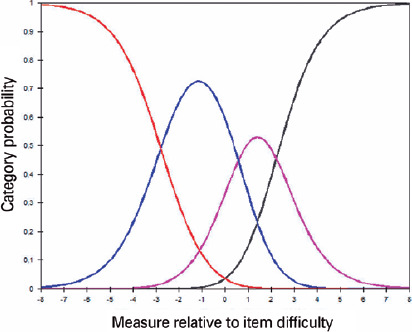
Category probability curves for the “difficulty in performing daily life activities in general” item.

The probability curves showed no evidence of threshold disorders (Figure 1); that is, the three thresholds of each item’s response categories were correctly ordered (-2.79; 0.57; 2.22 logit).

The reliability index of the people’s measurements (0.90) and the separation, PSI (3.07), were adequate, indicating that the questionnaire has measurement stability and good discriminatory ability.

### Person item map

**Figure 2:**
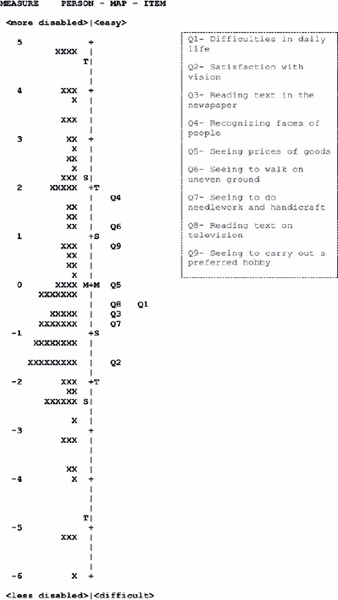
Map of the average calibration of the items and measurements of people in Catquest-9SF. Each participant (represented by the X symbol) is located to the left of the dashed line. Those with less severe disabilities are located at the bottom of the map. The items are located to the right of the dashed line.

People’s measurements (ability) and item calibration are graphically represented in the person item map (Figure 2). A very uniform item distribution is demonstrated. Item difficulty presented a 3.31 logit of dispersion (-1.61 to 1.70). The most difficult item was “satisfaction at the moment,” and the easiest item was “recognizing faces of people you meet.” The people’s abilities ranged from 11.32 logit (-6.56 to 4.76 logit; mean, -0.13), and the mean measurement (M) was similar to the mean calibration (M) of the items, which indicates good scale adequacy to the people’s ability level.

More difficult items are also located at the bottom of the map; M= mean; S= 1 standard deviation from the mean; T= 2 standard deviations from the mean.

## DIF

Item calibrations were compared between the sexes and between the two age groups (≤70 years vs >70 years). The differential item functioning (DIF)-contrast (item difficulty difference between the two groups compared, in logit) was relevant only in item 6, “seeing to walk on uneven ground” (e.g., female patients tend to consider themselves more satisfied with their ability to see obstacles than men, with a difference of 1.26 logit), and in item 2, “satisfaction with vision” (e.g., people aged >70 years tend to score as more dissatisfied with their vision than those aged ≤70 years, difference of 1.22 logit; Table 4).

**Table 4:** Differential item functioning (DIF) by sex and age

Description of the items	DIF by sex			DIF by age		
	Female calibration	Male calibration	Contrast	Calibration ≤70	Calibration >70	Contrast
Difficulties in daily life	0.7	-0.71	0.78	-0.34	-0.28	0.07
Satisfaction with vision	-1.58	-1.65	0.07	-2.25	-1.02	**-1.23**
Reading text in the newspaper	-0.43	-0.85	0.42	-0.41	-0.83	0.42
Recognizing faces of people	1.48	1.6	0.18	1.7	1.7	0
Seeing prices of goods	0.09	-0.29	0.38	0.04	-0.23	0.27
Seeing to walk on uneven ground	0.67	1.93	**-1.26**	1.49	0.98	0.51
Seeing to do needlework and handicraft	-0.93	-0.5	-0.43	-0.5	-0.94	0.45
Reading text on television	-0.15	-0.51	0.36	-0.44	-0.23	-0.21
Seeing to carry out a preferred hobby	0.46	1.14	-0.67	0.74	0.83	-0.09

### Correlation

The best-corrected visual acuity in logMAR with the best optical correction showed a statistically significant correlation with the Catquest-9SF logit score. A significant positive correlation was found between the two measurements (r=0.282 and p=0.004); that is, the variable visual acuity (LogMAR) increases while the questionnaire score also increases. Therefore, the greater the visual impairment, the higher the Catquest scale score.

## DISCUSSION

The results of this study confirm that Catquest-9SF was successfully translated into Portuguese and showed robust psychometric properties, making it suitable for use with Brazilian patients with cataracts.

The Brazilian Portuguese Catquest-9SF version is a one-dimensional, reliable, and valid questionnaire. In general, the infit and outfit MNSQ values showed an acceptable fit, considering the model expectation, and the PCA showed no other latent trait captured by the scale.

The presence of an outfit MSQN value >1.3 was observed in Table 3 (item 2, satisfaction with vision). After reviewing the data, we noticed that the value was influenced by the result of a single patient who had very high MSQN and ZSTD values (7.1 and 2.44, respectively). In this case, the patient reported having great difficulties in all aspects (maximum scores), but even so, he did not declare complete dissatisfaction with his vision, generating inconsistency in the overall response. When reviewing the characteristics of this patient in the database, we could not find a reason for this response pattern except for personal choice. The occurrence of this finding, however, does not influence the overall result of the analysis.

The score scale works well, with adequate score category progression (thresholds), mild mistargeting, and no significant DIF, corroborating the Swedish, Chinese, and Australian studies^(6,7,8)^.

The person item map showed a relatively uniform item distribution in the ability continuum. Although the average people’s measurement is close to the average item calibration, which suggests good targeting, three people had a minimum measurement, indicating that the questionnaire includes easy items for people with better vision. Similarly to other studies^(7,18)^, this study revealed that the most difficult item was “satisfaction with vision” and the easiest item was “recognizing faces of people you meet.” The general DIF assessment in this study showed that most items work similarly for participants with different sex and age characteristics. However, item 6, “seeing to walk on uneven ground,” presented a DIF in which women classified the item as easier than men, similar to the finding from Swedish and Australian studies^(6,7)^. Another DIF was found in item 2, “satisfaction with vision,” in which patients aged <70 years showed a lower level of satisfaction with their vision. This fact may be related to the greater visual demand of younger patients than older patients, with a tendency to increase complaints. We can infer that the higher prevalence of dementia-related diseases in older people may be a relevant factor in this case.

A previous study demonstrated that Catquest-9SF is sensitive in detecting changes caused by cataract surgery and can help identify patients eligible for surgery and estimate their improvement in the postoperative period^(11)^. Considering the specific characteristics of the Rasch model used for investigating the validity of this questionnaire (property of *objective specificity*), we can infer that the calibration of the Catquest-9SF items is not dependent on the sample (*sample free*) and does not need to be performed for each sample used^(15)^. Therefore, the results of the Portuguese version can be used for clinical purposes in the preoperative and postoperative periods, and their usefulness must be confirmed in future studies.

The limitations of this study are as follows: first, the lack of questionnaire response evaluation after the cataract surgery period; second, the possibility of a higher rate of dissatisfaction with vision in the patients evaluated due to the longer time interval between the cataract surgery indication and the time of the surgery in the Brazilian public health system; third, the low educational attainment prevalent in the population assisted by the Brazilian public health system, limiting the extrapolation of these data to the entire population; and fourth, the lack of information about the educational levels of the patients in this sample.

Finally, it is important to highlight that the use of questionnaires in Brazilian surgical practices for ecataract is often limited to research environments. The validation of a questionnaire with only nine items provides an easier and more viable daily application in clinical practice and can contribute to a more widespread adoption of this form of clinical assessment.

The Brazilian Catquest-9SF version presented evidence of validity and reliability, in addition to being linguistically and culturally understandable for Portuguese-speaking patients born in Brazil. It is an easy to understand and quick-to-use questionnaire that can adequately estimate the subjective visual functioning of patients with cataracts.
